# Predictors of Illicit Drug/s Use Among University Students in Northern Ireland, Wales and England

**DOI:** 10.5539/gjhs.v7n4p18

**Published:** 2014-12-16

**Authors:** Walid El Ansari, Lotte Vallentin-Holbech, Christiane Stock

**Affiliations:** 1 Faculty of Applied Sciences, University of Gloucestershire, Gloucester, United Kingdom; 2 Unit for Health Promotion Research, Institute of Public Health, University of Southern Denmark, Esbjerg, Denmark

**Keywords:** university students, illicit drug/s use, mental health, academic performance

## Abstract

**Introduction:**

The use of illicit drug/s among university students is a public health concern. Nevertheless, many UK studies investigated a narrow spectrum of variables to explore their association/s with illicit drug/s use.

**Methods:**

We assessed the associations between a wide range of socio-demographic, health and wellbeing variables (independent variables) and having used illicit drug/s regularly, occasionally or never in life (dependent variables). Data (3706 students) were collected from seven universities in England, Wales, and Northern Ireland, using a self-administered questionnaire.

**Results:**

About 5% of the sample had regularly used illicit drug/s, 25% occasionally, and 70% never. Regular drug use (RDU) was significantly more likely among males aged 21-29 years, daily smokers, those with heavy episodic drinking or possible alcohol dependency (CAGE test), and those who perceived their academic performance better than their peers. RDU was less likely among students with high health awareness and those living with parents. The predictors of occasional drug use (ODU) were similar to those of RDU. However, in addition, students with higher perceived stress were less likely, and students who felt financial burden/s were more likely to report ODU, while no association with academic performance was found. Never use of illicit drug/s was inversely associated with most of the variables listed above, and was positively associated with religiosity. Illicit drug/s use goes along with other substance use (alcohol and smoking). The finding that illicit drug/s use was higher among students reporting good academic performance was surprising and raises the question of whether illicit drug/s may be used as performance enhancing drugs.

**Conclusion:**

The factors identified with illicit drug/s use in this study could be utilized to develop appropriate public health policies and preventive measures for the health of students. Multilevel, value based, comprehensive, and strategic long-term intervention plans are required. This could include social interventions aimed at generating recreations alternatives and opportunities for youth, and a critical review for current authorities’ interventions and services. Suggestions for coping with problems of campus illicit drug use/abuse also need to be offered.

## 1. Introduction

The college years are a time of independence and separation from parental supervision. It is also a period characterized by transition, often precarious living conditions, dysfunctions of the education system and family pressure (Sommet et al., 2012). Thus these years provide opportunities to experiment with psychoactive substances (e.g. tobacco, alcohol, illicit drug/s), and are a phase in which substance use frequently increases (Locke et al., 2014). Students are at high-risk for substances and polydrug use (Maher, 2008; DROSICAN, 2009), and the initiation of or illicit drug/s use (IDU) amongst university students is evident globally (Mohammadpoorasl et al., 2014; Suerken et al., 2014; Sommet et al., 2012; Pickard et al., 2000; Bajwa et al., 2013; Maier et al., 2013; Gupta et al., 2013).

The undesirable outcomes of IDU among college students are a public health concern. These include unsafe sex (Mohammadpoorasl et al., 2014), sexually transmitted infections (Vivancos et al., 2008), poor academic performance (Bajwa et al., 2013), polydrug use (European Monitoring Centre for Drugs and Drug Addiction, 2009), depression, academic discontinuity and risk of post-college unemployment (Arria et al., 2013b). The relationship between IDU and academic performance is of interest to universities and colleges in terms of drug policies and prevention programmes. Whilst in Kuwait, drug use was positively associated with poor academic performance (Bajwa et al., 2013), in contrast, in Switzerland, students’ arguments for neuroenhancement drug/s included increased learning and increased performance (Maier et al., 2013). In Germany, the most common reasons for students’ stimulant use were to maximize time, increase motivation, and cope with memorizing (Hildt et al., 2014); and in Ethiopia, student’s expectation of khat was to help them in their study by staying awake/alert during long periods of studying and heavy academic work load (Kebede, 2002).

The types of illicit drug/s are numerous. Marijuana is a commonly used drug on campus, and is increasing (Suerken et al., 2014). Cannabis is frequently used (Pickard et al., 2000), where risky cannabis use has potential as a gateway drug leading to the use of more destructive illicit drug/s (Kandel & Jessor, 2002). Likewise, students regularly tried cocaine, crack, amphetamine, and inhalants (Stempliuk et al., 2005), and the pharmacological academic performance enhancement or cognitive enhancement via stimulant drug use and illicit stimulant use among university students is concerning (Hildt et al., 2014). Despite this, the majority of substance use research among college students has been on alcohol; more information is needed on prevalence, patterns, and correlates of IDU.

The risk and protective factors for IDU by university students are many. Among the variety of coexisting risk factors, being male, and the presence of other lifestyle characteristics and risk-taking behaviours e.g. smoking and alcohol drinking are the most common (Mohammadpoorasl et al., 2014; Suerken et al., 2014). Age/year of study also seem important, as experimentation with illicit drug/s increased among medical students in their second and fifth undergraduate years and after 1 year as house officers (Newbury-Birch et al., 2001); and in Ethiopia, students’ substance use had an upward increase among students from Year I to Internship program (Deressa & Azazh, 2011). As for financial burdens, students with a credit card debt of ≥ $1000 were associated with binge drinking and substance use (Nelson et al., 2008). Students’ IDU could also be related to mental health problems, as a way of coping with stress (Maier et al., 2013); or in relation to depressive symptoms, where students with depressive symptoms were at risk of interruptions in their college enrollment, and cannabis/heavy drinking added to this risk (Arria et al., 2013).

Conversely, among the protective factors, religion, living away from campus and/or living with their parents, and having better health and health awareness seem associated with lower IDU (Gomes et al., 2013; Mohammadpoorasl et al., 2014; Suerken et al., 2014). This wide range of risk/protective factors provided the rationale for the current study to examine many variables: socio-demographic (sex, age, year of study, living arrangements during university terms); health (self-rated general health, health awareness); lifestyle (illicit drug/s use, smoking, heavy episodic drinking, problem drinking, possible alcohol dependence); mental wellbeing variables (depressive symptoms, perceived stress), university related educational questions (academic achievement in comparison with peers), and information on religiosity and financial burdens.

The literature suggested knowledge gaps. First, few UK studies investigated university students’ illicit drug/s use (e.g. Pickard et al., 2000; Nixon, Youngson, & Beese, 2002; Barber & Fairclough, 2006; Underwood, Fox, & Manogue, 2010); and several studies seem outdated. Secondly, previous research had shortcomings: limited/modest sample sizes, and/or a narrow focus e.g. examined students from a single university, a single faculty, or even students from a single year (e.g. second-year medical students). Thirdly, many UK studies investigated a narrow spectrum of variables to explore their association/s with IDU. Such shortcomings are despite concerning UK evidence where 33.1% of students had IDU (Pickard et al., 2000), and Barber and Fairclough (2006) reported that 44% (dental students) and 52% (law students) had cannabis experience, with current use being 12% and 25% respectively; and a proportion of these students used other class A and B drugs e.g. ecstasy, amphetamines and cocaine.

The current study bridges these knowledge gaps to survey a generous sample of students (7 universities, different faculties) across England, Wales, and Northern Ireland, employing a range of associated variables, to describe lifetime prevalence of IDU, compare the variables’ bivariate relationships with IDU, and assess the variables associated with IDU (multifactorial analysis). Collectively, these features attach high importance to the study’s findings, and this study could be first to incorporate and mobilize many variables in order to understand the wider picture of illicit drug/s use across three UK countries.

The associations between three levels of lifetime IDU (regular, occasional, never) in relation to 4 sociodemographic variables (sex, age, accommodation during semester, financial burden/s), 2 general health variables (self-rated general health, health awareness), 4 mental health variables (depression, stress, sleep, religiosity), 4 other substance (tobacco and alcohol) use variables, and academic performance were examined. The specific objectives were to:

Describe selected sociodemographic characteristics of the sample and the lifetime prevalence of IDU;
Assess a range of variables by IDU status (regular, occasional, never) (bivariate analysis); and,
Assess and compare the variables associated with each IDU status (regular, occasional, never) controlling for all other variables (multifactorial analysis).

## 2. Materials and Methods

### 2.1 Sample, Procedures and Data Collection

The current UK data were part of a General Student Health Survey undertaken in many countries (El Ansari et al., 2007, 2011a, 2012a and b, 2013a and b, 2014a and b; Khalil et al., 2011). During 2007–2008, data (N = 3,706 students; 765 males, 2,699 females; mean age 24.9 ± 8.6 years) was collected at seven universities in three UK countries: England (Gloucestershire N = 908, Bath Spa N = 462, Oxford Brookes N = 203, Chester N = 883, Plymouth N = 167); Wales (Swansea N = 398); and the Republic of Northern Ireland (Ulster N = 463). Each participating institution provided ethical approval, and data were confidential/protected. Selection of the universities was premised on research interests and previous successful previous collaboration. A representative sample of students was sought at each site, and self-administered questionnaires were distributed to students attending lectures (last 10–15 minutes of the classes) of randomly selected courses at the universities. The sampling used for different universities was the same. Participation was anonymous and voluntary, no incentives were provided, and an information sheet attached to each questionnaire clarified the study objectives. Participants were informed that by completing the questionnaire, they agreed to participate in the study. Data were computer entered at one site to minimize data entry errors. Based on the number of returned questionnaires, the response rates were ≈80%.

### 2.2 Health and Wellbeing Questionnaire

The self-administered questionnaire gathered general health data: socio-demographic (sex, age, year of study, living arrangements during university terms); health (self-rated general health, health awareness); lifestyle (IDU, smoking, heavy episodic drinking, problem drinking, possible alcohol dependence); mental wellbeing variables (depressive symptoms, perceived stress), university related educational questions (academic achievement compared to peers), and information on religiosity and financial burdens. The tool was used and field-tested across many student populations (El Ansari et al., 2011a, 2012a and b, 2013a and b, 2014a and b). Variables with several response options were later dichotomized as shown in Table 2.

**Table 1:** Socio-demographics and lifetime illicit drug/s use of the sample

Variable	Lifetime Illicit Drug/s Use			

	Yes, Regularly N (Valid %)	Yes, a few times N (Valid %)	Never N (Valid %)	Total N (Valid %)
	168 (4.8)	886 (25.4)	2430 (69.7)	3484 (100)

Sex				
Female	83 (3.2)	583 (22.3)	1953 (74.6)	2619 (77.9)
Male	71 (9.6)	275 (37.2)	394 (53.2)	740 (22.1)

Age (years)				
18 - 20	64 (4.2)	356 (23.6)	1086 (72.1)	1581 (44.7)
21 - 29	69 (6.2)	352 (31.8)	687 (62.0)	1155 (32.7)
> 30	28 (3.6)	150 (19.5)	591 (76.9)	798 (22.6)

Year of study				
1st year	72 (5.0)	353 (24.5)	1014 (70.5)	1439 (42.4)
2nd year	51 (4.8)	273 (25.8)	736 (69.4)	1060 (31.3)
3rd year	38 (6.0)	172 (27.0)	427 (67.0)	637 (18.8)
4th year	2 (4.5)	18 (40.9)	24 (54.5)	44 (1.3)
≥ 5th year	0 (0.0)	4 (33.3)	8 (66.7)	12 (0.4)
Other	1 (0.5)	37 (18.5)	162 (81.0)	200 (5.9)

Financial burden/s				
No burden	94 (3.9)	552 (22.7)	1781 (73.4)	2427 (69.7)
Strong burden	74 (7.0)	334 (31.6)	649 (61.4)	1057 (30.3)

Accommodation during semester				
With parents	24 (2.8)	166 (19.2)	675 (78.0)	865 (24.8)
Not with parents	144 (5.5)	720 (27.5)	1755 (67.0)	2619 (75.2)

**Table 2:** Illicit drug/s use by selected variables among university students in the United Kingdom (Bivariate analysis)

Variable	Illicit Drug/s Use											

	Yes, regularly (n = 168)				Yes, a few times (n = 886)				Never (n = 2430)			

	N (%)	OR	*P*	95% CI	N (%)	OR	*P*	95% CI	N (%)	OR	*P*	95% CI
**Sex** (female)	83(3.2)	1.00			583(22.3)	1.00			1953(74.6)	1.00		
Male	71(9.6)	4.24	< 0.001	3.03-5.93	275(37.2)	2.34	< 0.001	1.95-2.79	394(53.2)	0.39	< 0.001	0.33-0.46

**Age** (18-20) years	64(4.2)	1.00			356(23.6)	1.00			1086(72.1)	1.00		
21-29	69(6.2)	1.70	0.003	1.19-2.43	352(31.8)	1.56	< 0.001	1.31-1.86	687(62.0)	0.63	< 0.001	0.53-0.74
> 30	28(3.6)	0.80	0.347	0.51-1.27	150(19.5)	0.77	0.020	0.62-0.96	591(76.9)	1.28	0.015	1.05-1.57

**Self rated general health** (Poor/Fair)	43(11.7)	1.00			105(28.5)	1.00			220(59.8)	1.00		
Good/very good/excellent	124(4.0)	0.29	< 0.001	0.20-0.42	762(24.8)	0.73	0.013	0.57-0.94	2186(71.2)	1.66	< 0.001	1.33-2.07

**Health awareness** (not at all/not much)	41(7.5)	1.00			164(29.8)	1.00			345(62.7)	1.00		
To some extent/very much	124(4.3)	0.51	< 0.001	0.35-0.74	703(24.4)	0.72	0.002	0.58-0.88	2057(71.3)	1.48	< 0.001	1.22-1.79

**Depressive symptoms** (low < 5th quintile)	120(4.2)	1.00			716(25.2)	1.00			20097(0.6)	1.00		
High (≥ 5th quintile)	48(7.5)	1.91	< 0.001	1.34-2.71	170(26.6)	1.13	0.215	0.93-1.38	421(65.9)	0.80	0.019	0.67-0.96

**Perceived stress** (< median)	77(4.1)	1.00			483(25.5)	1.00			1334(70.4)	1.00		
High (≥ median)	85(5.7)	1.43	0.029	1.04-1.96	381(25.4)	1.02	0.808	0.87-1.19	1032(68.9)	0.93	0.332	0.80-1.08

**Smoking** (never/occasional)	86(3.0)	1.00			664(23.0)	1.00			2139(74.0)	1.00		
Daily	80(14.8)	7.93	< 0.001	5.69-11.04	211(38.9)	2.71	< 0.001	2.21-3.32	251(46.3)	0.30	< 0.001	0.25-0.37

**Heavy episodic drinking** last 2 weeks (< 1 time)	28(2.8)	1.00			171(16.9)	1.00			812(80.3)	1.00		
≥ 1 time	124(6.0)	2.82	< 0.001	1.86-4.29	654(31.9)	2.44	< 0.001	2.02-2.95	1274(62.1)	0.40	< 0.001	0.34-0.48

**CAGE** score (< 2 positive responses)	96(4.0)	1.00			542(22.3)	1.00			1788(73.7)	1.00		
≥ 2 positive (**possible problem drinking**)	55(7.9)	2.70	< 0.001	1.91-3.83	260(37.5)	2.26	< 0.001	1.88-2.72	379(54.6)	0.43	< 0.001	0.36-0.51

**CAGE** score (< 3 positive responses)	126(4.4)	1.00			675(23.7)	1.00			2047(71.9)	1.00		
≥ 3 positive (**possible alcohol dependence**)	25(9.2)	3.39	< 0.001	2.12-5.40	127(46.7)	3.21	< 0.001	2.46-4.18	120(44.1)	0.31	< 0.001	0.24-0.40

**Importance of religion** in life (strongly or somewhat agree/neither agree nor disagree)	68(3.9)	1.00			380(21.9)	1.00			1291(74.2)	1.00		
Strongly disagree/somewhat disagree	90(5.7)	1.65	0.003	1.19-2.29	465(29.2)	1.53	< 0.001	1.30-1.79	1035(65.1)	0.65	< 0.001	0.56-0.75

**Financial burden/s** (Not at all/quite strongly)	94(3.9)	1.00			552(22.7)	1.00			1781(73.4)	1.00		
Strongly/Very strongly	74(7.0)	2.16	< 0.001	1.57-2.97	334(31.6)	1.66	< 0.001	1.41-1.95	649(61.4)	0.58	< 0.001	0.50-0.67

**Accommodation** during semester (not with parents)	144(5.5)	1.00			720(27.5)	1.00			1755(67.0)	1.00		
With parents	24(2.8)	0.43	< 0.001	0.28-0.67	166(19.2)	0.60	< 0.001	0.49-0.73	675(78.0)	1.75	< 0.001	1.46-2.09

**Academic performance** compared to peers (much worse/worse/same)	117(4.1)	1.00			703(24.8)	1.00			2010(71.0)	1.00		
Better/much better	45(8.5)	2.30	< 0.001	1.60-3.31	148(28.0)	1.26	0.033	1.02-1.56	336(63.5)	0.71	0.001	0.58-0.86

OR = odds ratio.

*Illicit drug/s use*: “Have you ever use/used drugs?” (‘Yes, regularly’; ‘Yes, but only a few times’; ‘Never’).

*Sociodemographic variables (3 items):* Age, sex and year of study at university based on self-reports. Age was categorized into three groups (18-20 years, 21-29 years, ≥30 years)

*Accommodation (living arrangements) during semester time*: “Where do you live during university/college term time?”, dichotomized into ‘living with parents’ *vs*. ‘not living with parents’.

*Financial burden/s*: “To what extent do you feel burdened in the following areas?” “Financial situation”, 1 = ‘not at all’, 6 = ‘Very strongly’, later dichotomized into 1, 2, 3, 4 = 1 *vs*. 5, 6 = 2 (strongly/very strongly).

*Religiosity (personal importance of religious faith)*: the extent to which participants agreed/disagreed with the statement: “My religion is very important for my life”, 1 = ‘strongly agree’, 2 = ‘somewhat agree’, 3 = ‘neither agree nor disagree’, 4 = ‘somewhat disagree’, and 5 = ‘strongly disagree’, later recoded into two categories based on agreement/disagreement (1, 2, 3 = 1 *vs*. 4, 5 = 2).

*Educational (academic achievement) variable*: The current study conceptualized and measured academic performance using students’ subjective comparative appraisal of their overall academic performance in comparison with their peers (El Ansari & Stock, 2010): “How do you rate your performance in comparison with your fellow students?” 1 = ‘much better’, 2=‘better’, 3=‘same’, 4=‘worse’, 5=‘much worse’, later dichotomized based on perceived better performance (3, 4, 5 = 1 *vs*. 1, 2 = 2).

*Self-rated general health*: “How would you describe your general health?” (1 = ‘poor’, 5 = ‘excellent’).

*Health awareness*: “To what extent do you keep an eye on your health?” (1 = ‘not at all’, 4 = ‘very much’).

*Depressive symptoms (20 items)*: using the Modified Beck Depression Inventory (M-BDI) (Beck et al., 1996; Schmitt et al., 2003). Sample items included: “I feel sad,” “I feel I am being punished,” “I have thoughts of killing myself,” “I have lost interest in other people,” “I have to force myself to do anything,” “I am worried about my appearance,” and “I have no appetite”. BDI computes a single score for individual respondents by summing their responses for all items of the scale. We used the 5^th^ quintile to categorize depressive symptoms as high.

*Perceived Stress Scale* (*4 Items*): Cohen’s Perceived Stress Scale (PSS) in its four item short form (Cohen, Kamarck, & Mermelstein, 1983) assessed the extent to which participants considered life situations to be stressful. PSS-4 is a simple psychological instrument that measures the degree to which situations in one’s life over the past month are appraised as stressful. The questions are general items designed to detect how unpredictable, uncontrollable, and overloaded respondents find their lives. All items began with: “In the past month, how often have you felt…?” (5 point scale: 0 = ‘never’, 1 = ‘almost never’, 2 = ‘sometimes’, 3 = ‘fairly often’, 4 = ‘very often’). In our sample, Cronbach’s alpha of PSS was 0.59. A median split (median = 11) categorized the variable into ‘Higher’ and ‘Lower’ stress (higher scores = more perceived stress).

*Smoking*: “Within the last three months, how often did you smoke? (cigarettes, pipe, cigarillos, cigars)” (daily, occasionally, never) (Hurrelmann & Kolip, 1994).

*Heavy episodic drinking* (*frequency*): “Think back over the last two weeks. How many times (if any) have you had ≥ 5 alcoholic drinks at a sitting?” [A “drink” is a glass/bottle/can of beer (≈50 cl), a glass/bottle/can of cider (≈50 cl), 2 glasses/bottles of alcopops (≈50 cl), a glass of wine (≈15 cl), a glass of spirits (≈5 cl) or a mixed drink] (Winther Ringgard et al., 2005). Responses were dichotomized into no heavy episodic drinking (<1 time) *vs*. heavy episodic drinking (≥1 time).

*Problem drinking* (*4 items*): A alcoholism-screening CAGE test (Ewing 1984) comprising 4 questions (Have you ever felt you should Cut down on your drinking? Have people Annoyed you by criticizing your drinking? Have you ever-felt bad or Guilty about your drinking? Have you ever had a drink in the morning to get rid of a hangover? (Eye opener). Each question is answered either “yes” or “no.” Two or more affirmative answers suggested problem drinking. We categorized respondents as non-problem (<2 positive responses) *vs*. problem drinkers (≥2 positive responses).

*Possible alcohol dependence* (*4 items*): ≥ 3positive CAGE responses (Ewing 1984) can suggest alcohol dependence. We categorized respondents as not possible alcohol dependence (<3 positive responses) *vs*. possible alcohol dependence (≥ 3 positive responses).

### 2.3 Statistical Analysis

SPSS v.22 was used for the statistical analysis (significance level set at 5%). To assess the different status of IDU, we stratified the dependent variable “have you ever use/used drug/s?” into three; ‘regular user *vs*. never user’ (N = 168), ‘occasional user *vs*. never user’ (N = 886) and ‘never user *vs*. ever user’ (N = 2430). Descriptive analyses for lifetime IDU and five socio-demographic characteristics were conducted for the whole sample and for each status of IDU. Using bivariate analysis the prevalence, odds ratio (OR) and 95% confidence interval (CI) for fourteen selected independent variables were calculated separately for each IDU status. In the multifactorial logistic regression analysis, we included university, age, gender and in addition, the eleven significantly associated predictors that emerged in the bivariate analyses. Several of the continuous variables were dichotomized (e.g. perceived stress, or BDI score for depressive symptoms) in order to interpret the results of the log regression models better. The OR for each drug use status was adjusted for; university, sex, age, self-rated general health, health awareness, depressive symptoms, perceived stress, smoking, heavy episodic drinking in the last 2 weeks, CAGE score, importance of religion, financial burden/s, accommodation during semester and academic performance compared to one’s peers.

## 3. Results

### 3.1 Sample Characteristics

The sample comprised 77.9% females and 22.1% males, with the majority of students being 18-29 years (77.4%) and at 1st or 2nd year at university (73.7%). A quarter of students lived with parents (24.9%), and a third reported financial burdens (30.3%). About 5% (3.2% females, 9.6% males) of the sample had regularly used illicit drug/s, 25% (33.3% females, 37.2% males) had occasional use, and 70% (74.6% females, 53.2% males) never used illicit drug/s in their life.

### 3.2 Variables Associated With Regular Illicit Drug/s Use (Bivariate Analyses)

Regular IDU was significantly more likely among male students aged 21-29 years, daily smokers, those with heavy episodic drinking and possible alcohol dependency (CAGE test), and among those who perceived their academic performance better than their peers (Table 2). In addition, regular IDU was less likely among students who had higher health awareness (kept an eye on their health), and among those living with their parents.

### 3.3 Variables Associated With Occasional Illicit Drug/s Use (Bivariate Analyses)

The predictors of occasional IDU were similar to those of regular drug use (Table 2). However, in addition, students with higher perceived stress were less likely and students who felt financial burden/s were more likely to use illicit drug/s occasionally, while no association with academic performance was found. Never using illicit drug/s was inversely associated with most of the variables listed above and in addition, was positively associated with religiosity (regarding religion as an important in my life).

### 3.4 Variables Associated With Never Use of Illicit Drug/s (Bivariate Analyses)

Table 2 shows overall positive associations between no IDU and female sex, good self-rated health, higher health awareness, being religious and living with parents. Students reporting no IDU were less likely to be aged 21-29 years, to report financial burden/s, report better academic performance than peers, have depressive symptoms, report heavy episodic drinking, problem drinking or possible alcohol dependence (score positive in CAGE alcoholism screening test), and to smoke.

### 3.5 Variables Associated With Illicit Drug/S Use (Multifactorial Analyses)

After controlling for all other variables (Table 3) some variables showed consistent positive associations with occasional and regular IDU, while exhibiting a negative association with never use: male gender, being aged 21-29 years, daily smokers, heavy episodic drinking, problem drinking or possible alcohol dependence (CAGE alcoholism screening test), and reporting financial burden/s. In addition, occasional and regular IDU were less likely, and never use was more likely among students who lived with their parents. The importance of religion (religiosity) was negatively associated with occasional IDU and positively associated with never using illicit drug/s. Better academic performance remained significant with regular IDU, but not with the other forms of IDU. Health awareness and perceived stress were not associated with regular IDU, but negatively associated with occasional use and positively associated with never use of illicit drug/s. Self-rated health and having depressive symptoms did not remain significantly associated with any form of IDU.

**Table 3:** Predictors of illicit drug/s use among university students in the United Kingdom (Multifactorial logistic regression analysis)

Variable	Illicit Drug/s Use								

	Yes, Regularly (n = 168)			Yes, a few times (n = 886)			Never (n = 2430)		

	OR	*P*	95% CI	OR	*P*	95% CI	OR	*P*	95% CI
**Sex** (Female)									
Male	**4.62**	<0.001	2.80-7.61	**1.86**	<0.001	1.46-2.38	**0.48**	<0.001	0.38-0.60

**Age** (18-20) years								
21-29	**1.65**	0.043	1.02-2.68	**1.90**	0.000	1.53-2.37	**0.54**	0.00	0.43-0.66
> 30	1.02	0.946	0.54-1.94	1.09	0.564	0.82-1.46	0.92	0.56	0.70-1.21

**Self rated general health** (Poor/fair)									
Good/very good/excellent	0.57	0.059	0.32-1.02	0.94	0.701	0.68-1.30	1.17	0.326	0.86-1.58

**Health awareness** (Not at all/not much)									
To some extent/very much	0.84	0.516	0.49-1.43	**0.71**	0.008	0.55-0.92	**1.39**	0.007	1.09-1.78

**Depressive symptoms** (< 5th Quintile)									
High (≥ 5th Quintile)	1.61	0.098	0.92-2.81	0.98	0.879	0.74-1.29	0.93	0.574	0.72-1.20

**Perceived stress** (Low < median)									
High (≥ median)	1.15	0.585	0.70-1.86	**0.79**	0.032	0.64-0.98	1.23	0.052	1.00-1.51

**Smoking** (Never/occasional)									
Daily	**11.73**	0.000	7.27-18.92	**2.48**	0.000	1.91-3.22	**0.32**	0.000	0.26-0.41

**Heavy episodic drinking** in last 2 weeks (< 1 time)									
≥ 1 time	**1.83**	0.021	1.10-3.07	**2.11**	0.000	1.67-2.67	**0.49**	0.000	0.39-0.61

**CAGE** score (< 3 positive responses)									
≥ 3 positive (**possible alcohol dependence**)	**1.94**	0.049	1.00-3.73	**2.20**	0.000	1.60-3.03	**0.46**	0.000	0.34-0.63

**Importance of religion** in life (Strongly or somewhat agree/neither agree nor disagree)									
Strongly disagree/somewhat disagree	1.16	0.499	0.75-1.80	**1.26**	0.024	1.03-1.54	**0.80**	0.022	0.66-0.97

**Financial burden/s** (Not at all/quite strongly)									
Strongly/very strongly	1.21	0.416	0.76-1.92	**1.35**	0.005	1.10-1.67	**0.74**	0.004	0.61-0.91

**Accommodation** during semester (Not with parents)									
With parents	**0.46**	0.011	0.26-0.84	**0.62**	0.000	0.49-0.79	**1.67**	0.000	1.33-2.10

**Academic performance** compared to peers (Much worse/worse/same)									
Better/much better	**1.88**	0.021	1.10-3.21	1.18	0.225	0.90-1.54	0.79	0.073	0.62-1.02

OR = odds ratio; Bolded cells indicate statistically significant odds ratio; OR adjusted for all other variables in the table and also for university.

## 4. Discussion

This study described the lifetime prevalence of IDU, and analysed the variables associated with regular, occasional, and never use of illicit drug/s among university students in the UK.

For the study’s first objective, we presented data from a relatively large sample across several institutions with a good geographical spread over the UK that have been anonymously collected with a high response rate and across a wide variety of academic/scientific disciplines. More than about one third of our students (30%) reported ever having IDU. Although we did not ask specifically for the type of illicit drug/s consumed, research suggested that cannabis or marijuana represent the drugs most often consumed among students in western societies, followed by amphetamines, ecstasy and cocaine (Vivancos et al., 2008; Newbury-Birch et al., 2001; Underwood et al., 2009).

To our knowledge, no recent universal surveys among UK students are available to compare our findings with. A smaller web-based survey among students from one UK university found 54% lifetime prevalence, but this sample was not necessarily representative (low response rate of 6%) (Vicancos, Abubakar, & Hunter, 2008). In another small UK study (136 medical students) (Pickard et al., 2000), a proportion of students (28% women, 36% men) similar to ours had IDU, but the time period of recall (e.g. ever, during the last month, etc.) is unclear. A representative report from the USA indicated that about the same proportion of American college students (37%) have ever IDU in their life (National Institute on Drug Abuse, 2010) as that observed in our study (30%). Similarly, in the USA (11 colleges), ≈ 30% of students ever had used marijuana, and 6% had other IDU at college entry; and among those who had never used marijuana prior to college, 8.5% initiated use during freshman year (Suerken et al., 2014). However, our observed 30% lifetime prevalence of any illicit drug/s seems moderate when compared several universities in Switzerland (44% reported lifetime IDU) (Maier et al., 2013); or when compared with the USA (119 colleges) (> 40% prevalence lifetime marijuana use) (Mohler Kuo et al., 2003). Of those who had any IDU in our sample, the majority reported having used illicit drug/s only a few times, but about 20% of users reported regular IDU (5% of the total sample). The proportion of regular users in our sample is in line with the 4% who had IDU in the past month in the Canadian National College Health Assessment (Kwan et al., 2013). Others have reported even higher proportions of regular IDU, although data are not directly comparable to ours (Arria et al., 2013b; Underwood et al., 2010).

In terms of the study’s other objectives, our bivariate analyses revealed a set of variables associated with IDU, and many of them remained significant in the multifactorial analyses. Males were almost twice as likely to be occasional users, and about 5 times more likely to have regular IDU than females, a pattern consistent with most other studies (Mohammadpoorasl et al., 2014; Vicancos, Abubakar, & Hunter, 2008; Newbury-Birch et al., 2001). While the gender difference is moderate regarding occasional IDU, for regular IDU, studies report a much higher consumption among males than among females (Underwood et al., 2009).

Regular and occasional IDU was significantly more likely among males, and among middle aged students (21-29 years old) as compared to younger students (<21 years), whilst students aged >29 years were not more likely to use illicit drug/s regulary or occasionally when compared with younger students (<21 years). It is not surprising that the lifetime prevalence increases with increasing age, and the fact that older students showed lower prevalences in our sample might indicate a cohort effect suggesting that experimenting with IDU has increased over the last recent years. A similar increase of IDU among college students has been observed in the USA (Mohler Kuo et al., 2010), the most recent European Drug Report also showed an overall stable but slightly increasing trend of the last year cannabis prevalence among young people in the UK between 2005 and 2011 (European Monitoring Centre for Drugs and Drug Addiction, 2013).

Independent of sex and other confounding factors, IDU among students was significantly associated with other substance/s use, e.g. daily smoking, heavy episodic drinking and high CAGE test scores (possible alcohol dependency). Such clustering of risk taking behaviours is in agreement with other studies of university students (Barber & Fairclough, 2006; Suerken et al., 2014; Mohammadpoorasl et al., 2014; Newbury-Birch et al., 2001). Specifically regarding combining alcohol with other drugs, in the USA, 87-98% of college users of marijuana or other drugs developed a heavy alcohol use pattern and many drink until they are drunk (Mohler-Kuo et al., 2003). Heavy drinking, smoking and drug use might be a hedonistic youth culture (Newbury-Birch, Walshaw, & Kamali, 2001), and using one drug lowers the barrier of taking another drug (Suerken et al., 2014). Alcohol intoxication increases the individuals’ risk of making ill-considered decisions about consuming illicit drug/s, thereby biasing the individual towards engaging in polydrug use (European Monitoring Centre for Drugs and Drug Addiction, 2009).

Regular IDU was less likely among those living with their parents, in agreement with Iran, where living in the ‘singles’ house in comparison to parental home was a risk for ever IDU (Mohammadpoorasl et al., 2014). In the USA, living on campus was associated with higher likelihood of initiating marijuana use during freshman year (Suerken et al., 2014); and across Germany, Denmark, Poland and Bulgaria, living in the parental home generally impacted positively on students’ lifestyle, e.g. was associated with healthy nutrition (El Ansari, Stock & Mikolajczik, 2012). Further, regular IDU was less likely among students who are more health aware (observe their health), which was not surprising.

Regular IDU was more likely among those who rated their academic performance better than their peers. In addition, students with higher perceived stress were less likely to use illicit drug/s occasionally. These findings indicated that higher IDU among students was associated with reporting better academic performance and with feeling less stressed. This suggests that students may use illicit drug/s intentionally to enhance their performance and/or to cope with stress. Indeed, perceived relief from psychological stress was among the most common reasons for alcohol, cigarettes, cannabis, or opiates use among students (Gupka et al., 2013); students who reported higher levels of performance pressure with regard to education, work, leisure, or family were also more experienced with neuroenhancing drugs (Maier et al., 2013), and students may cope by abusing performance enhancing drugs or other drugs, to deal with the academic stress (Mathew & Varga, 2012). Such findings raise concerns.

Our students who regarded religion as an important were less likely to having ever used illicit drug/s, in line with others who reported religiosity as protective against IDU (Mohammadpoorasl et al., 2014; Suerken et al., 2014). It is worth noting that we did not find an association between IDU and depressive symptoms. Pickard et al. (2000) similarly reported that neither the anxiety nor depression scales correlated with high levels of alcohol intake by students. Conversely, students with higher levels of depressive symptoms were more likely to use cannabis in an emotional pain or sex-seeking context (Beck et al., 2009). This might suggest that alcohol or illicit drug/s may be used for coping with depression for some students, but this does not seem to be the case in our sample, proposing that students’ IDU may be more a lifestyle component rather than a coping mechanism when feeling low.

This study has limitations. It is a cross-sectional study and no causal relationships can be derived. Self-reporting estimated the illicit drug/s use and biochemical and/or clinical validations were not undertaken. Our sample remains a convenience sample that invites caution with generalizations. Students with illicit drug/s use may be less likely to be present in class during the data collection, thus the currently observed prevalence of illicit drug/s use may under-estimate the true prevalence in this population. Despite these limitations, the study has important strengths as to the best of our knowledge, no previous study seems to have investigated in detail the relationships the prevalence, and correlates of regular and occasional illicit drug/s use and the associations between such use and a wide range of variables across large samples of students from many different faculties within a variety of universities in the United Kingdom.

## 5. Conclusion

The factors identified with IDU could be utilized to develop appropriate public health policies and preventive measures that may improve the health of student populations. Especially the finding that IDU is highly associated with alcohol misuse and smoking calls for action related to preventing risk taking behaviour in general. The study provides also support for initiatives directed towards male students and those with low health awareness, as illicit drug use was highest in these groups. Multilevel, value based, comprehensive, and strategic long-term intervention plans are required that consider the risk and protective factors. These could include social interventions aimed at generating recreations alternatives and opportunities for youth, and a critical review of current authorities’ interventions and services. Motivational interviewing interventions should target the primary substance whilst considering other multiple risky substance use behaviours. Suggestions for coping with problems of campus illicit drug use/abuse also need to be offered.
